# The impact of the COVID-19 pandemic on the health behaviours of people living with and beyond breast, prostate, and colorectal cancer—a qualitative study

**DOI:** 10.1007/s11764-022-01234-8

**Published:** 2022-07-19

**Authors:** Caroline Buck, Simon Pini, Phillippa Lally, Rebecca J. Beeken, Abigail Fisher

**Affiliations:** 1 UCL Department of Behavioural Science and Health, University College London, 1-19 Torrington Place, London, WC1E 7HB UK; 2 Leeds Institute of Health Sciences, University of Leeds, Level 10 Worsley Building, Clarendon Way, Leeds, LS2 9NL UK

**Keywords:** Cancer survivorship, Health behaviours, COVID-19, Pandemic, Qualitative, Typology

## Abstract

**Purpose:**

Positive health behaviours (sufficient exercise, healthy diet, limiting alcohol, and not smoking) can improve multiple outcomes after a cancer diagnosis. Observational studies suggest that health behaviours were negatively impacted for some but not all individuals living with and beyond cancer. The aim of this study was to qualitatively explore the impact of the pandemic on health behaviours of people in this population.

**Methods:**

Thirty participants were purposively sampled for characteristics including diagnostic group (breast, prostate, and colorectal cancers), gender, time since diagnosis, and age. Semi-structured interviews were conducted to discuss the impact of the pandemic on health behaviours. Thematic analysis and a secondary Ideal Types analysis were conducted.

**Results:**

Five themes covered changes in food, weight management, relationship to alcohol, and exercise. Five “types” were identified, representing orientations to health behaviours. The “gift of time” provided by the pandemic had an impact on health behaviours, with trends towards increases in drinking, eating unhealthy food, and exercising less.

**Conclusions:**

The COVID-19 pandemic impacted engagement in positive health behaviours among participants in this study. Strict restrictions and changes in routines resulted in individuals adjusting how they managed their diet, alcohol intake, and exercise behaviours. The typology identified within this study helps to define how different orientation to health behaviours could underpin the responses of individual people LWBC.

**Implications for cancer survivors:**

Alongside providing an understanding of the experiences of people LWBC during the COVID-19 pandemic, the proposed typology suggests how, with further development, future health behaviour interventions in this group could be targeted based on individual orientations to health, rather than demographic or clinical variables.

**Supplementary Information:**

The online version contains supplementary material available at 10.1007/s11764-022-01234-8.

## Background

Incidence rates for all cancers have increased in the UK, with breast, prostate, and colorectal cancers accounting for more than 40% of diagnoses [1]. Whilst cancer prevalence is rising, early detection and treatment advances have resulted in reduced mortality rates, meaning more people are living with and beyond cancer (LWBC) [2]. However, people LWBC often suffer disease and treatment-related sequelae that negatively impact quality of life such as weight gain [3, 4], anxiety and depression [5], sleep disturbances [6], pain [7], and fatigue [8]. A large body of evidence suggests that engaging in positive health behaviours can help diminish many of these side effects [9–14], so much so that the World Cancer Research Fund (WCRF) and the American Institute for Cancer Research (AICR) recommend people LWBC should aim to be physically active for at least 150 min per week, maintain a moderate weight, consume a healthy diet, restrict energy dense foods, reduce consumption of red meat, avoid smoking, and limit alcohol intake [15].

In March 2020, the COVID-19 pandemic necessitated radical modifications to the activities of the general population, leading to a significant reduction in physical activity (PA) and alterations in eating habits [16, 17]. This is of concern to people LWBC, given that before the pandemic began the proportion of this population attaining WCF/AICR guidelines was already low [18, 19]. Changes in health behaviours among this population have been observed during the last 18 months but have been inconsistent. For example, a cross-sectional epidemiological study among women with breast cancer demonstrated that previously active participants decreased PA and gained weight during lockdowns [20]. Similarly, two studies on the impact of restrictions to the delivery of face-to-face PA programmes for people LWBC showed more people were physically inactive in the period after the onset of the pandemic compared to before it. Participants cited difficulty in accessing exercise opportunities, decreased motivation, reduced fitness levels, and increases in weight as barriers to engage in PA [21, 22]; however, the same studies found that more motivated individuals LWBC maintained or even increased PA during lockdowns. A qualitative study among eighteen women with breast cancer found that some participants reported how new habits such as yoga, Pilates, gardening, and walking helped to retain PA levels during the restrictions, but that many participants also reported weight gain and consequent deleterious effects on health attributed to reduced PA levels [23].

Research has also shown inconsistent changes in alcohol intake during the pandemic amongst the general population. A continuous cross-sectional study comparing pre- and post-pandemic drinking behaviour during the first period of lockdown in the UK found no significant change in consumption or drinking frequency in Scotland, and a fall in consumption in England [24]. Conversely, a study investigating individual-level changes in alcohol intake and behaviour among six thousand adults showed overall increases in the number of days where alcohol is consumed across genders and ages, with more women reporting increased incidence of heavy drinking episodes compared to men [25]. In opposition again, a continuous cross-sectional survey among over two thousand participants found that older adults were more likely to demonstrate notable increases in alcohol intake compared to younger counterparts [26].

Given that quantitative survey data on the effects of the pandemic on health behaviours of people LWBC are inconsistent (and in some areas limited), it is critical to explore the challenges faced by this population in greater detail to determine who has been negatively affected, what support they may need during and beyond the pandemic, and how they could be supported should future pandemics occur. Qualitative research can help explore and unravel conflicting findings through the exploration of divergent perspectives, and the observation of how people deconstruct and conceptualise health and illness, helping to examine attitudes, barriers, and motivations that are not always accessed through numerical data [27, 28]. At time of writing, qualitative research exploring the impact of the pandemic on people LWBC has focussed on patients undergoing cancer treatment, the impact of the pandemic on cancer services, and the psychosocial effect of the pandemic on people with cancer [e.g., [29, 30]. To the best of our knowledge, there is no qualitative work on the health behaviours of people LWBC during the pandemic, aside from the aforementioned breast cancer study [24]. Therefore, the aim of this study was to qualitatively explore the impact of the COVID-19 pandemic on the health behaviours of people LWBC.

## Methods

### Design

This qualitative study used constructivist epistemology to explore the impact of the COVID-19 pandemic on the health behaviours of people LWBC. This epistemology is consistent with the aim of investigating how participants understood their experiences at the time of the interview, whilst acknowledging this was a snapshot in time created in conjunction with the researcher.

### Participants

Participants were enrolled in the Advancing Survivorship After Cancer: Outcomes Trial (ASCOT), a randomised controlled trial of a habit-based health behaviour intervention [31]. ASCOT participants were recruited between 2015 and 2019. To be eligible for ASCOT, participants had to be adults (aged ≥ 18 years), self-reporting a diagnosis of non-metastatic breast, prostate or colorectal cancer, not be receiving active anti-cancer treatment (except for oral anti-cancer treatments taken at home), and be able to understand spoken and written English.

As part of a COVID-19 follow-up, participants completed a quantitative survey and were asked for their consent to be approached for a qualitative interview about health behaviours during the pandemic. When recruitment to the qualitative study began in December 2020, 620 participants had consented to be approached. This provided a large pool of potential participants that allowed for purposive sampling based on demographics characteristics. Sampling characteristics included age, gender, diagnosis, geographical location, marital status, ethnicity, and ASCOT experimental group allocation (intervention or control). Ethical approval for the COVID-19 follow-up was granted by South Central–Oxford Ethics Committee in September 2020 as an amendment to the original ethical approval for ASCOT.

### Data collection

Selected participants were approached via email or telephone, and one-to-one interviews were conducted by telephone using a semi-structured interview schedule (see Appendix A), which used open questions to explore broad categories of health behaviour including diet, exercise, physical health, alcohol consumption, sleep, and quality of life. Interviewers were guided by the participants wherever possible and explored “off-topic” content for potentially rich, novel and unpredictable information. Interviews were audio recorded and transcribed verbatim for analysis, with identifiable information removed.

### Analysis

A flexible thematic analysis was employed as the guiding analytical principle, as recommended by Braun and Clarke [32, 33]. Six stages of thematic analysis were followed: 1) familiarisation; 2) coding; 3) searching for themes; 4) reviewing themes; 5) defining and naming themes; 6) writing up.

During the first four stages, emerging ideas and points of interest were discussed in regular research team meetings. Unique cases and interview excerpts were explored to assess how they interacted with current emerging ideas. This process continued until themes were thought to accurately represent the experiences of participants. The authors (SP and CB) then labelled themes in stage 5, before fully coding all transcripts to finalise the framework. During writing up, the thematic framework was further scrutinised and refined.

### Ideal types

Whilst conducting the thematic analysis, specific “types” were identified within the sample which appeared to reflect certain orientations and approaches to health behaviours, and underpinned the way in which participants appeared to respond to the challenges of the pandemic. Therefore, a secondary methodology—Ideal Type analysis [34]—was conducted to identify, classify, and describe participants’ behavioural patterns whilst allowing the totality of their behaviour to be explained [35]. Ideal Types analysis harnesses a critical realist perspective for examining parallels and disparities between phenomena and identifying patterns of participants with similar approaches. Seven stages are followed when conducting this analysis: 1) ‘Becoming familiarised with the data set’, 2) ‘Writing the case reconstructions’, 3) ‘Constructing the Ideal Types’, 4) ‘Identifying the optimal cases’, 5) ‘Forming the Ideal-Type descriptions’, 6) ‘Checking credibility’, 7) ‘Making comparisons’ (see Appendix B for more details).

## Results

### Participants

From the 620 potential participants, an initial 10% shortlist was created based on the sampling characteristics. From this shortlist, a total of 32 participants were approached to take part in the interviews as they presented with the diversity of sampling characteristics required. Two individuals declined to participate, explaining their reasons as current physical illness and depression. Thirty participants were interviewed, after which thematic saturation was attained; therefore, the rest of the shortlist was not approached. Sample characteristics are shown in Table 1. The most prevalent characteristics were white-British (75%), married (70%), and aged 50–70 (77%).

**Table 1:** Participant characteristics

	*N*	%
Total	30	100
Gender		
Female	16	53
Male	14	47
Age-group		
39–49	4	13
50–59	11	37
60–69	12	40
70 +	3	10
Diagnosis		
Breast	10	33
Prostate	10	33
Colorectal	10	33
Marital status		
Married/living with partner	21	70
Single	6	20
Divorced	3	10
Living location		
City	12	40
Village	9	30
Town	7	21
Hamlet or isolated dwelling	2	6
Ethnicity		
White British	22	75
White other	2	6
Black Caribbean	3	10
Black African	2	6
Asian	1	3
Time since most recent diagnosis (years)		
Under 6.5	4	13
6.5–7.5	11	37
7.5–8.5	10	33
Over 8.5	5	17

Interviews were conducted November 2020–February 2021. To provide context for the results reported in the subsequent sections, an overview of the cycles and stages of lockdown in the UK is provided in Table 2.

**Table 2:** Cycles of COVID-19 lockdown (March 2020–February 2021)

March	The UK goes into the first national lockdown
April	Lockdown is extended in combination with a set of recommended targets for the government to meet before it is lifted
May	Plan for conditional easing of lockdown, including return to work for those who cannot work from home
June	Phased reopening of schools and non-essential shops. Easing of two-metre social distancing rules, but then implementation of local lockdowns by the end of the month
July	Local lockdowns applied to more areas, with others allowed to reopen certain businesses (pubs, restaurants, hairdressers). Local authorities given more powers to make local decisions
August	Further easing of lockdowns with encouragement for the public to go back out to restaurants /other leisure facilities
September	“Rule of six” introduced for social gatherings. Return to working from home and a 10 pm curfew introduced
October	Introduction of the “tier” system for local lockdowns, followed by the second full national lockdown
November	Second national lockdown in force
December	Many places given highest “tier 4” status, but restrictions relaxed for the Christmas period to allow minimal gatherings
January	Third full national lockdown
February	Publication of “roadmap” to systematically lift restrictions across the UK

### Thematic framework

Five overarching themes were identified which applied predominantly to dietary and exercise behaviours: 1) changes in the role, importance, and meaning of food; 2) weight management; 3) drinking behaviour and relationship to alcohol; 4) adapting following disrupted exercise behaviours; and 5) motivation for exercise and maintenance of routines.

#### Changes in the role, importance, and meaning of food

Lockdown measures triggered significant shifts in all aspects of daily life and routine. Some participants reported changes in the procurement of food as the pandemic developed, with initial panic buying resulting in specific shortages (bread, certain fresh fruit, and vegetables). A proportion of the sample—particularly those who felt at risk with comorbidities, were experiencing bouts of illness, and those deemed ‘vulnerable’—avoided food shops as the lockdown unfolded. Some reacted to the loss of agency by drastically narrowing their food/meal choices, whereas others appeared to become more adventurous, recognising they had to “make do” with what was available or rely on delivery companies for some fresh food.

There’s a little bit of an unknown of what’s turning up….I literally looked at this green thing and wondered what it was, I mean it was a squash!

Many participants noted the uniqueness of having an unspecified amount of time with no plans or routine, with some dubbing the experience as the ‘gift of time’, which appeared to initiate a cascade of changes in relation to how food was considered, consumed, and reflected upon. With cancelled holiday plans and an unexpectedly hot spring/early summer, some participants felt able to relax and actually ‘enjoy’ early lockdown, and food took on a central role. This was particularly true of those living with family in more rural settings with outdoor space, who were able to relax and spend time together as a unit, almost as if they were ‘on holiday’. However, the juxtaposition of a quasi-celebratory atmosphere set against the increasing bleakness of the news appeared to lead to changes in the types and frequency of food eaten among many participants. Restrictions meant that people moved less and ate more, preparing and consuming richer, more indulgent meals and snacks in greater volume, especially at the start of the pandemic. Food appeared to act as a panacea for a number of emotions, ranging from fear, boredom, commiseration and anxiety, to euphoria, excitement and celebration.

We were eating very indulgently; it was as if it was Christmas so whatever we wanted to buy we were buying

The nature of participants’ original cancer diagnosis appeared to play a role in the motivation of some to retain dietary habits during the pandemic, where relationships with food had been altered after diagnosis and a particular diet followed.

Cancer showed me that I wasn’t infallible….mortality situation and what happens if you don’t look after yourself

This was particularly prevalent among participants with a colorectal cancer diagnosis, many of whom were acutely aware of the link between diet and its impact on their bodies. The presence of a stoma added a layer of complexity to this relationship, with a daily reminder of the impact of too much or the wrong kind of food.

You’re more connected than you’d ever believe…if I indulge myself in something too much I know what the consequences are

Other participants reported their habits did not significantly change during the pandemic; some had kept a food diary since diagnosis and tried hard not to veer from it. Information from GPs, the internet and friends/family were reported as influencing food choices and behaviours.

Overall, the early phase of the pandemic provoked anxiety for a number of participants. For many, this initial difficulty with the fundamental need to obtain food exacerbated feelings of uncertainty and distress, which resulted in a focus on food that seemed to endure throughout the pandemic.

#### Weight management

Awareness of weight was an important issue for many participants and one that came into sharper focus for some as the pandemic progressed and early indulgences persisted. Weight issues described by participants fell broadly into three groups:

i) moderate weight gain; this was reported mostly by women with colorectal cancer diagnoses and/or participants living alone, some with significant comorbidities and less family/social support. Emotional difficulties, loneliness, and pre-pandemic weight issues were reported to have contributed to the weight increase;
ii) minor weight gain; mostly reported by women with breast and colorectal cancer diagnoses. This group cited the changes in routine enforced by restrictions, the weather and the ‘holiday’ atmosphere at the beginning of the pandemic for decreasing PA and reduced resistance to sweet/sugary snacks. There was also evidence that the gain/loss of the same few pounds was a normal behaviour over the course of a year under non-pandemic circumstances, where a small weight increase acted as a trigger to reduce calorie intake and increase exercise;
iii) stable weight or minor weight loss; reported mostly by older men (most of whom had a prostate cancer diagnosis) and women who had a somewhat inflexible approach to food/health—expressed through keeping food/weight diaries and sticking rigidly to a certain regime. Two women in this group reported that they had experienced eating disorders in the past.

A change in eating habits was reported by a number of participants across the three groups after the summer ended, which coincided with worsening infection rates and subsequent lockdowns. This more conscious and cautious approach to food and eating appeared to be underpinned by a growing awareness of the link between metabolic risk factors and death rates from COVID-19, as these facts increasingly entered the vernacular.

We’d sit and play games….nibbling crisps….we looked back and thought it was gorgeous but it all had consequences which as sensible people we knew we had to change

A number of participants reported attempts at resetting their eating habits; reducing frequency of baking and eating desserts, cooking healthier recipes, decreasing meat-based meals, and for some, reinstating tried and trusted methods of food restriction and weight loss (i.e. Weight Watchers, Slimming World). Support from family members was deemed important in this endeavour; for some, a collective/family attempt at healthier habits was implemented. This was less straightforward for those living alone and/or with significant comorbidities and reduced access to shopping and cooking, with some single participants reporting staying healthy was very challenging throughout the pandemic, as boredom and despondency increased, and food appeared to act an antidote. Many reported increasing reliance on convenience food as shopping opportunities were limited, entering into a cycle of eating energy dense foods that became difficult to resist.

#### Drinking behaviour and relationship to alcohol

The ‘gift of time’ posed a challenge for participants in the management of their alcohol intake. Whilst pre-pandemic baseline alcohol intake varied across the sample in terms of drinking frequency, type of alcohol chosen, and number of drinks consumed in one sitting, many participants described increases during the pandemic.

It’s a little bit of ‘actually do you know; I can’t really do anything else!’

Many participants noted they drank more frequently, consumed alcohol earlier in the day than normal, and opted for more indulgent drinks (e.g. swapped beer for cocktails, drank gin and tonics instead of white wine). Some reported ‘treating’ themselves with alcohol, or compensating for the absence of pleasure elsewhere; alcohol as a ‘reward’ was commonly raised as justification for this behaviour.

I often do dry January, but this year I thought…‘oh god life’s tough enough’....

Some participants noted whilst they were drinking more frequently at home, consumption was more evenly spread across the week, rather than drinking higher volumes at the weekend in pubs and restaurants. A number of people used this change in habit as a justification for higher intake, i.e. they felt that ‘slow and steady’ vs ‘binge’ amounts kept their intake at an acceptable level.

Fewer participants professed to reducing alcohol consumption as summer ended and winter approached (as many had to some extent with food choices), although ‘Dry January’ did appear to prompt some to consider their behaviour around alcohol a little more closely. A small group of participants reported drinking more alcohol led to them snacking more on salty, fatty foods, and exercising less. With Christmas cited as a normal time for indulging in alcohol, the bad weather, dark evenings, and ever-rising flow of bad news provided a convenient backdrop for sustained increases, suggesting a growing reliance for some on the mood changing quality of alcohol. For some single, urban dwelling participants, reliance on alcohol was often cited.

It’s been quite easy to reach for the bottle on these gloomy evenings

In contrast, a contingent of participants reported the pandemic did not change their alcohol consumption at all. For some, their original diagnosis had resulted in a reduction in, or even elimination of alcohol, which did not change in lockdown.

No, I stopped drinking oh about three years ago, I said it’s not worth it now so I’ve not taken a drink in about three years

A number of participants cited an intolerance to alcohol since treatment/surgery (esp. those with colorectal cancer); others reported their cancer diagnosis prompted a realisation of the link between alcohol and ill health (particularly women with breast cancer).

#### Adapting following disrupted exercise behaviours

The pandemic resulted in substantial reductions in exercise behaviours for many participants, with closures of sports clubs, gyms, pools, and other facilities. Some participants also described reducing physical housework and gardening because of a lack of visitors. Having to stop work, or change to working from home, was difficult for those with physical jobs who missed the exertion of their work. Even those in more sedentary employment lost the opportunity to exercise on their commute or at lunchtimes.

at work, I go up one, two, three, four, five, six, seven, about eight flights of stairs.

Regular exercise activities prior to the pandemic were reported as presenting a structure and routine to people’s lives, providing motivation to get through other challenging parts of their week. As these activities had to be abandoned because of the pandemic, some participants described the loss of other routines they had attached exercise to, such as swimming after a school run, or walking following a day at work.

I’d do the swimming, depending on what time my shift started or I’d do it after if I hadn’t got to do a school pick up or drop off, or something. I’d go and have a swim and a sauna, and stuff like that after work

Participants often described the absence of exercise activities in combination with other losses. Many who paired socialising with exercise found it hard to replicate this type of social activity during the pandemic. Without the common link of the exercise/sport/club, it appeared difficult to maintain those specific social relationships. Others missed the sense of personal wellbeing they felt from their exercise activities and reported an associated reduction in their general feelings of vitality and motivation.

physically I feel like I’ve aged…because you’re not doing anything physical, and I’m used to doing something physical at least two or three times a week, you miss that and it then …ages you

Those participants who reduced PA complained of feeling less tired yet wearier, with decreased sleep quality and tendency for daytime naps. The opposite was claimed by those who increased PA levels in lockdown.

I actually think it makes me more tired …I sit here and think ‘how the hell did I have the energy to go dancing’ and then I think … doing exercise …gives you that bit more energy

Whilst some participants struggled to exercise during the pandemic, many attempted to embrace the ‘gift of time’ by replacing lost activities and routines with something new. They described using the extra time to more easily plan and structure alternative activities. The majority engaged in regular walking, often describing it as ‘the only thing I can do’, but there were many other examples of home gyms/circuit training, running, cycling, and online classes. Although more accessible, some participants (particularly older) found the move to online exercise was ‘just not the same’ and clearly lacked comparable social benefits.

#### Motivation for exercise and maintenance of routines

The motivation to exercise and maintain this activity was described as being driven by internal factors, external influences, and environment. Good weather facilitated outdoor exercise during early phases of lockdown, whereas restrictions during the winter months appeared to be more challenging, with little to do to offset the sense of loss of freedom. This restricted participants’ ability and willingness to engage in healthy behaviours, often substantially reducing or discontinuing activities and routines previously developed during the early stages of the pandemic.

Some factors, such as the desire to be competitive or to maintain a certain body image, appeared to be driven by how participants saw themselves in relation to others, which provided an ongoing calibration of their success. Others were more driven by their inner sense of health and wellbeing, such as the links between exercise endorphins and good mental health or maintaining levels of fitness. This was often reinforced by the news, which spoke increasingly of the link between health and susceptibility to severe COVID-19. Several participants discussed longer term goals based on wanting to be healthy and ensure a good quality of life as they aged.

after the [cancer] hit you were realising that life could end and therefore, I didn’t want to have an unhealthy older age

A number of participants reported how the exercise behaviours of family members, friends, or other cancer survivors inspired them to increase activity and try new things. Additionally, the influences of health professionals (GPs, oncology staff) and YouTubers in recommending appropriate activities were also cited as sources of inspiration and motivation. Being at home during the pandemic had variable impact on motivation for participants, with many citing that greater flexibility with their time facilitated new activities, whereas others found ‘it’s not so easy to motivate yourself at home’.

Being able to set targets was often a motivating factor for maintaining exercise behaviours and provided meaning and purpose to the activities. Externally prescribed targets, such as 10,000 steps or preparing for specific challenges and events, were motivation for some, whereas others set their own personal targets to work towards.

I use a Fit Bit now and I would have only have done about 2,000 steps a day, whereas now I do a lot more, 8,000-10,000 steps

Monitoring progress provided a structure and metric for success for some, which was managed through apps, spreadsheets, or simply by noticing improvements in fitness. However, some participants found that setting—and then missing targets—had the opposite effect on their enthusiasm.

A contingent of participants had more successful maintenance when exercise was integrated into other activities or had a particular purpose. Location was an important factor, with many discussing the importance of having easy access to open spaces to exercise in. The weather was a significant factor and could be used as a positive reason to go outside when it was pleasant, and an excuse to stay indoors when it was inclement. For most participants, social restrictions meant that a primary motivational factor was being outdoors and just being ‘desperate to do something’.

#### Ideal Types analysis

A secondary analysis to investigate health behaviour typology produced five types. The intention of this analysis was to describe amplified or optimal forms of the types present within the data [35]. Participants appeared orientated to one of these types above the others, although a minor degree of overlap between types was observed. (See Appendix C for more detailed descriptions and example quotes).

#### Long-term habitual exercisers

Reasonably health aware, with regular PA perceived as an important and pleasurable activity comprising a meaningful part of their lives. Monitoring PA was deemed satisfying and valuable, and motivation to undertake it appeared driven by a strong internal mechanism prompting regular participation; ‘an inner monologue saying ‘right you need to do something now’… (it) wants me to do the right thing’. Exercise was often prioritised over other activities in their day, its importance seemingly part of their self-concept; ‘It’s who I am …it’s what I do’. Many participants in this group also had a pragmatic and balanced approach to their diet, claiming to adhere to healthy guidelines. However, their ‘inner monologue’ was less present in relation to alcohol; a good number of individuals used their adherence to PA/dietary guidelines to justify their alcohol intake.

#### Pragmatic integrators

Most in this group did not concern themselves with dietary, PA, or alcohol guidelines beyond basic principles (i.e. not smoking, less red meat, some fruit and vegetables). Participants expressed the importance of ‘staying active’, which was deconstructed as ‘being on the move’, being able to walk reasonable distances, being active in their work, not sitting down for too long at any one time and keeping a positive attitude in life through hobbies and social interaction, as opposed to formally seeking to undertake PA. Some retired individuals reported their grandchildren kept them ‘on their toes’, and believed gardening and housework were ways of ‘keeping fit’. The notion of gym membership or pursuit of formal exercise as separate to everyday activity was not part of their agenda. Prior to the pandemic, some in this group tended to work in manual jobs (commercial kitchen worker, supermarket lorry loader, brewery worker), and all felt their jobs were sufficiently physically demanding to reassure them that their activity level was acceptable. Part of this reassurance appeared to be predicated on a comparison between themselves and others who were deemed less active.

#### Reactive convertors (cancer diagnosis)

This group was characterised by its shift in attitude towards health behaviours after a cancer diagnosis. Prior to cancer, participants claimed they had varying degrees of negative health behaviours, a lack of awareness of healthy guidelines, and a disinterest in and/or inability to action change in regard to their health. This was despite comorbidities such as diabetes, high blood pressure, high cholesterol, and advice from medical professionals to tackle these problems. The cancer diagnosis triggered a marked awareness of their own responsibility for their wellbeing. However, this was moderated by the perceived seriousness of their diagnosis. For example, some women with a breast cancer diagnosis and certain men with prostate cancer, whose disease allowed a reasonably straightforward treatment (e.g. lumpectomy only or a ‘watch and wait’ approach respectively) reported a feeling that they ‘didn’t have ‘proper’ cancer’ compared to those with a more complex disease pathway. Some participants cited how schemes such as local NHS intervention-based programmes targeting weight and PA gave them a realistic starting point combined with gentle goal setting, which felt manageable and not overwhelming. In some cases, participants in this group appeared to start displaying similar characteristics to the Long-Term Habitual Exercisers, but with the motivation stemming from their diagnosis.

#### Inadvertent convertors (pandemic)

Unlike Reactive Converters, this group included people whose cancer diagnosis appeared not to have triggered changes in health behaviour. However, the notion of responsibility for their own health began to develop specifically during lockdown, through a combination of serendipity, the ‘gift of time’, and a growing awareness of the link between health and susceptibility to Covid-19. For some, unplanned events such as being gifted a Fitbit or being inadvertently enrolled in a diabetes prevention programme by their GP contributed to triggering health behaviour modifications. This resulted in changes during the pandemic that included seeking out health advice (friends, family, internet), an examination of activity levels and a reappraisal of dietary and alcohol habits. Growing knowledge and participation was seen as empowering, and many in this group reported increases in physical and mental well-being as they progressed on their pandemic-inspired journey. It seems feasible that the changes initiated in this group might be more fragile given they were linked to transitory events that did not carry the same impact as the Reactive Converters; therefore, the potential to ‘return to normal’ could be higher in this group.

#### Health aware but unable/resistant to change

The smallest group in the sample, participants here were characterised by their age (generally younger), greater severity of cancer diagnosis/treatment and more substantial presence of comorbidities. Two subgroups were evident: 1) participants who recognised engaging in positive health behaviours was important and claimed to want to make changes, but felt unable to do so because of fatigue, mobility issues from injury/overweight, complications from cancer treatment (e.g. stoma) and an acknowledgment their eating/drinking patterns might be disordered/addictive (i.e. overeat, binge eat or drink, do little or no exercise); and 2) participants who expressed an understanding of the associated risks of negative health behaviours but felt it was equally important to eat and drink what they want and live their lives to the full. This was largely because they had been diagnosed early and felt life was for living rather than adhering to guidelines; ‘It was like do you know what, I’m alive so eat what you want’. Some participants across both sub-groups reported loneliness and an absence of social/family support that appeared to impact their eating, drinking and exercise behaviour.

## Discussion

The COVID-19 pandemic had an impact on the ability of people LWBC to engage in healthy behaviours. The ‘gift of time’ that arose from lockdown restrictions had a variable impact on participants, with some able to attend to positive aspects of their lives, and others finding this more challenging, especially as the restrictions extended for an uncertain duration; the pandemic has shown health behaviours can be fragile and it can be difficult for some people to adapt to changes in routine. However, there were some positive adaptations in the sample, with many participants demonstrating resilience, good intentions, and an ability to accept and adapt to changes.

Recent research has shown the pandemic has impacted on the health behaviours of the general public, with evidence of reduced PA, worsening dietary habits, and increased alcohol intake, behaviours that are linked with deleterious health consequences [16, 17, 25, 26]. Given the specific needs of people LWBC and the fact their levels of PA and adherence to a healthy diet were suboptimal before the pandemic began, the importance of healthy behaviours in this population is particularly pertinent, as evidence links negative health habits to higher risk of metabolic changes, disease recurrence, and all-cause/cancer specific mortality [10–12]. As such, it is important that the long-term effects of disruptions to eating, drinking, and exercise routines caused by the pandemic should be monitored in people who are currently LWBC.

Participants in this study described the restrictions caused by the pandemic as having an impact on their ability to engage in healthy behaviours in all areas. In keeping with previous literature, participants with a breast cancer diagnosis did appear to be more vulnerable to increases in sedentary behaviour and weight [3, 4]. Participants across the diagnostic groups described some increases in alcohol consumption, which was consistent with previous research [26]; however, this seemed to be particularly significant for those living alone. The nuances of changes in relationship to alcohol were important. Those participants experiencing the ‘holiday’ feeling of the early pandemic seemed able to reset their intake more easily as time went on, but others citing ‘boredom’ or ‘reward’ found this more difficult. The fact that alcohol intake appeared to increase for many participants in this sample, even those with more entrenched positive health behaviours in other areas, is noteworthy.

Analysis of participant accounts demonstrating reduced PA because of restricted access to normal routines supports previous findings, with more motivated individuals finding alternatives and less motivated participants struggling more [21, 22]. Existing health behaviours appeared to rely substantially on routines, and the typology started to show that those participants whose routines derived from internal mechanisms found it easier to adapt than those whose behaviours were externally motivated and organised. However, beginning to uncover typology related to health behaviour orientation in people LWBC has shown that even habitual exercisers struggled at times and needed support. A pre-pandemic population-based cohort study examining differences in key health behaviours between a general population sample and people LWBC found no significant differences, suggesting a cancer diagnosis does not provoke lasting behaviour change [36]. However, the typology reported in this current study suggests that orientation to health behaviours may be substantially influenced by diagnosis experiences for some types of people LWBC. Given the importance of healthy lifestyles, strategies for effective support for behaviour change in people LWBC need to be identified, especially as enduring behaviour change is minimal in this group [18, 19, 36]. The typology may provide a useful starting point for the design of future interventions, which could identify and target support for people based on specific attitudes and orientation towards health behaviours, rather than demographic or clinical characteristics. For example, a Long-Term Habitual Exerciser may require advice on specific metrics to monitor their exercise, or strategies put in place to adjust their existing activities to current circumstances or limitations. They might also benefit from advice regarding alcohol intake. Pragmatic Integrators may need simple guidelines to follow that easily fit into their everyday lives and do not demand too much from them. Reactive Converters would feasibly need advice and information specifically linked to their cancer diagnosis and long-term effects. Inadvertent Converters may respond well to an initial intervention, but may require more frequent contact and prompting to maintain health behaviours. They may also benefit from a variety of different schemes at different times to keep things novel and interesting. The Health Aware but Unable/Resistant to Change group would need their specific barriers identified and addressed where possible, but would also likely benefit from a flexible approach that does not prescribe specific targets.

Participants in this study described varying feelings towards their cancer diagnosis, with attitudes appearing to be influenced in part by perceptions of its ‘seriousness’ and arguably by health behaviour orientation. Some participants claimed that they did not particularly feel as if they had ‘real’ cancer, especially if their treatment was straightforward. It is feasible that these attitudes could have an impact on health behaviours and go some way to explain why a cancer diagnosis does not easily prompt enduring health behaviour shifts in some people. It may be that being discharged following treatment could represent an important ‘teachable moment’ [37] to assess a person’s individual orientation to their health behaviours, reinforce the importance of sustained good habits after recovery from cancer, and design a suitable plan for how they could manage their health when living beyond cancer.

## Conclusion

The COVID-19 pandemic imposed challenging restrictions on the extent to which people LWBC could engage in positive health behaviours. Loss of routines provided an increase in unstructured time, which resulted in difficulties for many in managing their diet, alcohol intake, and exercise behaviours. The typology identified within this study has begun to define how different orientation to health behaviours could explain the responses of individual people LWBC. Future research could look to explore these typologies further and develop an assessment tool that could evaluate individual health behaviour orientations in practical terms, which has the potential to inform the way health behaviour interventions are targeted to people LWBC in the future.

## Strengths and limitations

The methodology employed in this study was predominantly participant-led, rather than theoretically driven. This allowed our participants the opportunity to lead the direction of the interviews as much as possible and therefore determine how they described their health behaviours. This is a strength of our data collection in terms of understanding participant experiences, but perhaps also a limitation through reduced emphasis on exploring theoretically informed topics.

Although every effort was made to recruit a diverse sample of participants broadly representative of the population of those LWBC, the final sample was slightly skewed towards white-British, economically stable, married people. This was determined to some extent by the distribution within the overall ASCOT sample, which is a trial sample and reflective more of the population who take part in trials than the population of people LWBC. We acknowledge our findings and the conclusions we draw may not be generalisable to the general population; it is feasible that individuals who agree to participate in health behaviour research might have greater motivation for health behaviour change than the average person LWBC [38]. However, this study was conducted some years after participants had signed up to the original ASCOT trial [31] and could simply reflect a desire to help others and contribute to scientific research [39] rather than participating for their own gain.

The interview methodology was based on subjective self-reporting of health behaviours. Whilst allowing for important participant-led constructions of experiences, this method did mean it was difficult to be certain of the accuracy of what was described. Although every effort was made to ensure participants felt comfortable, there is a possibility that social desirability and anxiety about discussing alcohol intake, weight, diet, and exercise may have influenced what was reported.

The typologies work presented in this paper was not the initial primary focus of the study. The interviews did not explicitly explore typology and therefore there were possible areas of rich data that were not part of data collection. The conclusions drawn about typologies are preliminary and require further exploration and development.

## Supplementary Information

Below is the link to the electronic supplementary material.

Supplementary file1 (DOCX 28 KB)

## Data Availability

The code manual including definitions, code rules, and anchor codes, generated during the current study is available from the corresponding author on reasonable request.
