# Supplementary material for: The impact of the COVID-19 pandemic on the health behaviours of people living with and beyond breast, prostate, and colorectal cancer—a qualitative study
Source: J Cancer Surviv. 2022 Jul 19;17(5):1488–98. doi: 10.1007/s11764-022-01234-8 (PMC9296113; doi:10.1007/s11764-022-01234-8)
Supplement: Supplementary file 1 — Supplementary file1 (DOCX 28 KB) [file 11764_2022_1234_MOESM1_ESM.docx]

**APPENDICES**

Appendix A – Interview Schedule

*NB: The researcher will have access to the participant’s questionnaire answers during the interview. Before they begin they will look over the responses and will therefore know the participant’s employment situation and living arrangements as well as their comorbidities.*

*Introduce yourself.*

*Ask permission to record the conversation. Ask them not to use any names so that the recording is anonymous. Then turn recorder on.*

Thank you so much for completing our questionnaire for us. We are collecting some really interesting data. The purpose of today’s call is to allow you to tell us in more detail about how things have been for you this year.

I’ll start with a very general question and then we can perhaps get into more details as we go on if that’s OK. What are the main ways that the coronavirus pandemic has impacted your life?

*Let them answer this however they choose. If they don’t cover the below topics then ask them about them:*

- Did you stay at home during lock-down? Did you go out at all? Have you started going out or having people to visit more now?
- Has the coronavirus pandemic had any impact on your health *(check if they report having had coronavirus before asking this and if so focus on that first) (consider the health conditions they mentioned in the questionnaire and changes to medical care, as well as any reported changes to how they responded to experienced symptoms)?If they say it has then discuss their feelings about this.*
- Has the pandemic resulted in you changing any of your health behaviours, for example your diet or exercise? *Let the participant lead this to start with and discuss all the behaviours they mention having changed. Also look at the answers to qu 51 and discuss any behaviours that they reported change on that they have not spontaneously mentioned.*
- Do you think you will be changing your health behaviours in the coming months?
- Do you feel that your quality of life has improved or worsened over the course of this year? *(consider sleep, loneliness, fatigue, health).*
- Do you think your quality of life will change over the coming months?
- *If they answered anything other than agree and strongly agree to question 57 on the questionnaire (implying that they are not sure or would not be willing to have a COVID-19 vaccination) ask they why they answered as they did and what their reasons are.*
- Is there anything else that you would like to tell us about?
- Do you have any questions?
- *Thank them for their time.*

Appendix B – Ideal Type Analysis Stages

**Step 1**: ‘Becoming familiarised with the data set’ was achieved by ensuring the lead authors conducted all the interviews themselves, listened to the audio files and read/reread the transcripts.

**Step 2**: ‘Writing the case reconstructions’ was achieved by creating a depiction of the data for each participant (‘case’) interviewed. These were primarily descriptive in nature and included researcher interpretation plus initial attempts at identifying key aspects of each participant’s experience/perspective.

**Step 3**: ‘Constructing the Ideal Types’ was achieved by the systematic comparison and contrast of the case reconstructions with each other, the aim being to explore similarities and differences between cases in order to identify patterns and form groupings (Ideal Types) of similar cases.

**Step 4**: ‘Identifying the optimal cases’ was achieved by identifying a single case reconstruction per ideal type which best illustrates the essence of that type in an optimal form. Gerhardt (1994) describes this as an almost overdrawn account of each pattern.

**Step 5**: ‘Forming the ideal-type descriptions was achieved by constructing a detailed illustration that represents each case and thus characterising the ideal type to which it belongs.

**Step 6**: ‘Checking Credibility’ was achieved through discussion and consensus building with another researcher (who had not been part of the analysis process) in order to evaluate the clarity of the ideal types and check that the interpretations were predicated on the data.

**Step 7**: ‘Making comparisons’ was achieved through focusing on whole cases and on the patterns that exist between the cases, in order to establish why specific cases have been grouped in a specific ideal type at the same time as capturing the disparity in experiences and insights of the cases within each ideal type.

Appendix C – Idea Types in more detail & example quotes

**Long-term Habitual Exercisers**

*Ideal type description*

These participants characterised themselves as being reasonably health aware, with regular physical activity (PA) comprising an important part of their lives. Most described how they had become involved and interested in exercise from an early age, either engaging with a specific sporting interest or simply coming from an ‘active/sporty’ family that helped to foster healthy habits. For these individuals, PA is seen as an important and often pleasurable activity that doesn’t require effortful participation. For some, motivation to undertake it appeared to be driven by a strong internal mechanism prompting regular participation; “*an inner monologue saying ‘right you need to do something now’… (it) wants me to do the right thing*”. Exercise appears to be habitual and ingrained into their routines, often prioritised over other activities in their day.

All were keenly aware of the benefits of regular PA in providing physical and psychological well-being during and after participation, as well as its role in protecting their health and mobility for later years; “*I want to preserve the loveliness of life*”. The group broadly comprised individuals who pursued either ’solitary’ PA e.g., gym members, regular joggers, cyclists, swimmers, and those whose exercise involved a more ‘social/shared’ experience e.g., tennis/badminton players, regular dance class and yoga/Pilates enthusiasts. Use of exercise trackers was high in this group, with many citing the need to achieve a minimum of 10,000 steps a day. Others (usually older/male) recorded their exercise activity in writing, finding this activity both satisfying and valuable; “*It helps me keep track*”. Several individuals across both exercise types stated they were competitive on a personal basis (‘can I jog further/walk more steps than last time?’) and with others (‘I want to win this match’). Rational for this behaviour included the need to improve and strive towards continued fitness, and the importance placed on PA among some participants suggests it might comprise part of their self-concept; “*It’s who I am …it’s what I do*”.

Alongside PA, many participants in this group had a reasonably pragmatic and balanced approach to their diet, claiming to adhere to healthy guidelines such as limiting red meat, sugar and unhealthy fats and increasing fibre/fruit and vegetables. For some individuals, the same internal mechanism governing PA was said to be present for weight maintenance, where a gain of a few pounds triggered a strict regime to take back control and lose it. This inner monologue was less present in relation to alcohol in this group; a good number of individuals used their observance of PA/dietary guidelines to justify alcohol intake, which sometimes was claimed to be far from ideal.

*Impact of the pandemic*

For individuals who pursued solitary PA such as jogging and cycling, the pandemic appeared to have little impact, given that these activities were permitted during lockdown (although some participants admitted that they probably broke the rules to run/cycle further from home than was allowed). Because of the closure of all leisure/organised sports centres during lockdown, social sport enthusiasts and gym members fared less well, as participants reported how the closure impacted them given their exercise habits were predicted on groups/clubs. For many, the loss of social contact tied up with their chosen PA was deemed difficult. A number of participants appeared to replace their habitual routine/sport with something different e.g., purchased home fitness equipment and set up a mini-gym at home, took up jogging instead of dancing, or tried to do yoga/Pilates via Youtube. Others (esp older) found the adjustment difficult to cope with and did not substitute their usual activity with anything else; “*I can’t play tennis, I can’t play bowls….I don’t know what else to do*”. Given the importance of PA in their lives prior to the pandemic, some participants reported feeling less well, experiencing lowered mental acuity and diminished enthusiasm because of reduced activity. A number of participants in this group also reported concomitant negative dietary changes (more snacking, bigger portions) and increased alcohol intake during the various lockdowns.

*In their own words*

*“I do a fifteen minute walk before I do anything else in the morning…it’s a good start to the day”*

*“24 days a month I do it (jogging) and I’ve been doing it forever and a day”*

*“doing moderate or a reasonable amount each day allows you to continue to do it indefinitely”*

*Keywords*:

**Inner directed, ingrained, goal-oriented, steadfast, future proofer, balancer, committed, knowledge seekers/gatherers**

**Pragmatic Integrators**

*Ideal type description*

This group tended to comprise older participants living in a traditional marriage paradigm. Participants characterised their approach to PA as knowledge of the importance of ‘staying active’ as opposed to formally seeking to undertake PA separately. ‘Staying active’ was deconstructed as being on the move, able to walk reasonable distances, not sitting down for too long at any one time and keeping a positive attitude in life through hobbies and social interaction. Most in this group tend to work (or used to work) in manual jobs e.g., commercial kitchen worker, supermarket lorry loader, and brewery worker, and all felt that their jobs were sufficiently physically demanding to reassure themselves that their activity level was acceptable. Part of this reassurance appeared to be predicated on a comparison between themselves and others who were deemed less active. Some retired individuals reported that their grandchildren ‘kept them on their toes’, and that gardening and housework were their way of staying active. The notion of gym membership or pursuit of formal exercise ‘*for the sake of it’* was not part of their agenda.

From a dietary perspective, this group tended to be fairly traditional eaters, enjoying British dishes such as egg and chips, fish fingers and sausages (although most individuals stated that they knew they ought the frequency of intake of the latter). Men in this group were largely uninvolved in food decision making, purchasing and preparation, relying on the traditional construct of their wife undertaking these responsibilities. An overall approach to health behaviours in this group is one of unawareness and pragmatism, most not worrying too much about diet, PA or alcohol, and certainly not appearing to take too much advice to heart besides basic guidelines (no smoking, cutting back on red meat, ensure some fruit and vegetables etc).

*Impact of the pandemic*

Given the main source of activity was their employment, participants who were furloughed or lost their jobs during the pandemic reduced their activity almost overnight. This resulted in some people gaining weight amid an increasing sequence of inactivity, boredom and comfort eating. Gardening was cited as assisting in offsetting some of this inactivity, and short walks to and from the local shops served to kill time and notch up some movement. However, the weather was often used as a reason for non-adherence to any outdoor activity, indeed a number of participants confessed that the colder months led them to disengage with the outside world and reduce activity further with the loss of social contact and visitors at home or outside. Combining this with a number of age-related comorbidities, individuals in this group appeared at risk of very low activity levels and more negative eating behaviours during the pandemic.

*In their own words*

*“We haven’t been anywhere other than my wife does the shopping…it’s been really a case of sticking indoors, sticking in the house…this latest lockdown has been difficult with the weather and the general miserable feeling everywhere”*

*“This year because I’ve not been as active as I usually would be, my weight has gone up a bit. And that doesn’t do the hip joint and things like that any favours”*

*Keywords*:

**Traditional/conventional, set in their ways, unaware, fair-weather, easily distracted**

**Reactive Convertors (Original cancer diagnosis)**

*Ideal type description*

This group is characterised by its shift in attitude towards health behaviours after cancer diagnosis compared with before. Prior to cancer, participants reported varying degrees of negative health behaviours, a lack of awareness of healthy guidelines, and a disinterest in and/or inability to action change in regard to their health, despite comorbidities such as diabetes, high blood pressure and high cholesterol. The advent of a cancer diagnosis triggered a marked change in health habits as awareness of their own responsibility for their health dawned. This understanding tended to be inspired by medical staff e.g., GPs, oncology clinicians, allied health professionals and advice from the internet. Particularly useful was patient enrolment in local NHS intervention-based programmes targeting weight and PA organised by their GP. Guidelines, education and support were cited as integral to the success of these programmes, leading some participants to become inadvertent habitual exercisers and adherers to positive dietary habits and reduced alcohol intake. Some participants cited how a realistic starting point combined with gentle goal setting felt manageable and not overwhelming, leading to outer directed benefits such as weight loss and improved body image.

Other individuals described how their pursuit of renewed health encouraged a journey to find a form of PA that suited them, often trying different activities and programmes to get a good fit. Whilst not always evangelical, they recognised the value of positive health behaviours and the physical and psychosocial benefits derived from being more health aware. A number of individuals in this group admitted that disruption to their new routines (weather, ill health, family responsibilities, inertia etc), might derail their positive health behaviours, suggesting a conflict between this new stance and their ‘*old self’* which could result in barriers to re-engagement in PA and other health behaviours.

*Impact of the pandemic*

Some participants in this group reported how the ‘gift of time’ afforded to them during the pandemic allowed for renewed efforts to improve dietary habits and increase PA during the lockdowns. Participants described having increased time to push themselves to achieve greater fitness goals; “*I could cycle for miles and miles*”. However, like Long-term Habitual Exercisers, a contingent struggled with the closure of organised activity and faced an overnight loss of activity, which in some cases led to a loss of routine and uptake of previously entrenched negative health behaviours such as overeating and greater alcohol intake.

*In their own words:*

*“I think having been through that time it sort of makes you realise what good health is and to appreciate it”*

*“After the train hit (diagnosis) you were realising that life could end and therefore I didn’t want to have an unhealthy older age”*

*Keywords:*

**Health epiphany, require education/support, future proofer, teachable, potential lapsers**

**Inadvertent Convertors (Pandemic)**

*Ideal type description*

Unlike diagnosis-related Reactive Converters, this group included people whose cancer diagnosis appeared not to have triggered changes in health behaviour. This may be due to a perception that their cancer was ‘not serious’ (e.g., men with prostate cancer who have been told they would likely ‘die with cancer rather than of it’, or women who had a lumpectomy and no subsequent treatment for breast cancer; “*I didn’t have real cancer*”). It is also feasible that the impetus to make health behaviour changes had not dawned or could not be supported by circumstance, knowledge, motivation and opportunity.

However, through a combination of the ‘gift of time’, a growing awareness of the link between health and health behaviours (as evidenced by daily pandemic statistics and reports of the disproportionate risk of overweight, obesity, inactivity and metabolic comorbidities in COVID-19 deaths) and for some, a fortuitous event such as being gifted a Fitbit or being inadvertently enrolled in a diabetes prevention programme by their GP, the notion of personal responsibility for their own health began to develop during lockdown. This resulted in some changes in health behaviours that included seeking out health advice (friends, family, internet), an examination of activity levels and a reappraisal of dietary and alcohol habits. Some participants discussed how YouTube provided them with a wealth of information and guidance, leading to trying out exercise regimes and participation in free ‘classes’ at a time suited to them. Increasing knowledge and participation was seen as empowering, and many in this group reported increases in physical and mental well-being as they progressed on their pandemic -inspired journey.

*Impact of the pandemic*

Whilst lockdown and the gift of time appeared to initiate health behaviour improvements, the question arises as to whether previous unhealthy behaviour habits will return once life returns to normal following the restrictions imposed during the pandemic.

*In their own words*

*“(Before the pandemic) I wouldn’t nearly worry so much about having a balanced diet and exercising on a regular basis so I would say it has had a big impact in that respect”*

*“I had a lot of plans what to do but then it (lockdown) came to an end because I had to go to work”*

*Keywords*:

**Health epiphany, (short term) habitual exercisers, teachable, enthusiasts, potential lapsers**

**Health aware but unable/resistant to change**

*Ideal type description*

The smallest group, participants here were characterised by their age (generally younger), greater severity of cancer diagnosis and presence of more comorbidities. Two sub groups were evident: 1) participants who recognised that engaging in positive health behaviours was important and claimed to want to make changes, but felt unable to do so because of fatigue, mobility issues from injury/overweight, complications from cancer treatment (e.g., stoma) and an acknowledgment that their eating/drinking patterns might be disordered/addictive (i.e. overeat, binge eat or drink, do little or no exercise); and 2) participants who expressed an understanding of the associated risks of negative health behaviours but felt it was equally important to eat and drink what they want and live their lives to the full, largely because they had been diagnosed early and felt that life was for living rather than adhering to guidelines; “*It was like do you know what, I’m alive so eat what you want”.* Some participants in this group reported loneliness and an absence of social/family support that appeared to impact their eating, drinking and exercise behaviour.

*Impact of the pandemic*

All in this group claimed that the pandemic had a negative effect on their health behaviours, as they fell prey to decreased PA, increased drinking and eating energy dense food. One participant explained that she attempted intermittently to take control and embark on a healthier regime, only to be thwarted fairly quickly and give up.

*In their own words*

*“If I walk five minutes I have to stop and take breaths”*

*“When I’m stressed I comfort eat … I eat biscuits, cheesy crisps…I’ll eat like 3 or sometimes even six (packets)”*

*“Because of lockdown … I’m indoors, I’ve gained a lot of weight”*

*Keywords*:

**Inactive, addictive, vicious circle, defined by comorbidities, needs support and supervision**
